# Associations of meditation with telomere dynamics: a case–control study in healthy adults

**DOI:** 10.3389/fpsyg.2023.1222863

**Published:** 2023-07-14

**Authors:** Nirodhi Namika Dasanayaka, Nirmala Dushyanthi Sirisena, Nilakshi Samaranayake

**Affiliations:** ^1^Research Promotion and Facilitation Centre, Faculty of Medicine, University of Colombo, Colombo, Sri Lanka; ^2^Department of Anatomy, Genetics & Biomedical Informatics, Faculty of Medicine, University of Colombo, Colombo, Sri Lanka; ^3^Department of Parasitology, Faculty of Medicine, University of Colombo, Colombo, Sri Lanka

**Keywords:** telomere length, telomerase, gene expression, *hTERT*, *hTR*, meditation

## Abstract

**Introduction:**

Telomeres are protective end caps of chromosomes which naturally shorten with each cell division and thus with age. Short telomeres have been associated with many age-related diseases. Meditation has come to the fore as a mind–body practice which could influence the telomere dynamics underlying these phenomena. We previously reported meditation to be associated with higher telomerase levels, mindfulness and quality of life. Here, reporting on the same study population, we describe associations between long-term meditation and telomere length (TL), expression of *hTERT* and *hTR* genes and methylation of the promoter region of *hTERT* gene.

**Methods:**

Thirty healthy meditators and matched non-meditators were recruited. TL was measured using quantitative PCR, gene expression was assessed using reverse transcriptase PCR, and methylation level was quantified by bisulfite-specific PCR followed by Sanger sequencing. Comparisons between meditators and controls were carried out using t-tests, while Pearson correlation was used to identify correlations, and regression was used to identify predictors.

**Results:**

Males comprised 63.4% of each group with an average age of 43 years. On average, they had meditated daily for 5.82 h (±3.45) for 6.8 years (±3.27). Meditators had longer relative TLs (*p* = 0.020), and TL decreased with age (*p* < 0.001) but was not associated with other socio-demographic variables. Regression analysis showed that age (*p* < 0.001) and duration of meditation (*p* = 0.003) significantly predicted TL. The meditators showed higher relative expression of *hTERT* (*p* = 0.020) and *hTR* (*p* = 0.029) genes while the methylation level of the promoter region of *hTERT* gene was significantly lower when compared to non-meditators (*p* < 0.001). Negative correlations were identified between the methylation level of the promoter region of *hTERT* gene and the expression of the *hTERT* gene (*p* = 0.001) and duration of meditation (*p* = 0.001).

**Conclusion:**

The findings suggest that meditation as a lifestyle practice has multi-level beneficial effects on telomere dynamics with potential to promote healthy aging.

## 1. Introduction

Telomeres are (TTAGGG)_n_ simple repeat DNA sequence tracts that serve as protective end caps of linear chromosomes. They are typically 5 kb to 15 kb in length in somatic cells (Blackburn et al., 2015). Telomere length (TL) reduces with each cell division due to progressive loss of these tracts. Uncapping and the loss of telomeres can lead to cellular senescence, apoptosis and genomic instability. Lengthening of the telomeres is carried out either enzymatically by telomerase or by a recombination based mechanism (Blackburn, 2005). Telomerase activity is dependent on the expression of two main genes; *TERT* (or *hTERT*) which encodes the core protein sub-unit and *TERC* (or *hTR*) which encodes the RNA component. Both are indispensable components of telomerase activity (Pawlas et al., 2016). However, specific genetic regions collectively referred to as promoters and enhancers, are regulators of gene expression. The regulatory activities at a molecular level can be influenced by multiple factors, including DNA methylation, histone modification, and DNA looping (Doane and Olivier, 2017). DNA methylation is driven by the addition of a methyl radical to a deoxycytosine, which is typically found in cytosine-phosphate-guanine (CpG) sites (Richardson, 2003). CpG rich sites accompanying transcriptional sites are identified in the promoter regions of genes. CpG island methylation of the *hTERT* gene is known to be linked to silencing of the gene (Rebekah et al., 2007).

Moreover, it has been suggested that healthy lifestyle factors including physical exercise (Arsenis et al., 2017), body mass index <25 kg/m^2^ (Gielen et al., 2018), non-smoking (Sulastri and Lestari, 2017) and a healthy diet (Paul, 2011) can enhance telomere maintenance. Mind and body interventions such as meditation, yoga, Tai-Chi, and Qigong have also been recognized as healthy lifestyle factors that affect telomere regulation. Meditation refers to a range of techniques; for instance, loving-kindness, body-scanning, walking, breathing, focused attention and mindfulness meditation. While operationally meditation can be practiced *via* two methods: either focusing attention on a changing object such as physical perceptions of the body including pain, temperature, and pressure or focusing attention on a static/repetitive object such as breathing (Esch, 2013), producing an emotionally calm and stable mind, relaxation, tranquility and self-awareness are some of the common end points of meditation (Horowitz, 2010). Further, the increasing body of evidence on the beneficial effects of meditation on mental and physical health in addition to emotional health, has led to approaches incorporating meditation-based techniques such as Mindfulness-Based Stress Reduction (MBSR) being adopted widely in allopathic health care settings. These findings and practices have, in turn, also led to studies which explore the underlying molecular mechanisms which bring about such beneficial changes. One such area of expanding interest is the association of meditation with cellular aging where the length of the telomeres may vary due to the effects of meditation. In addition, meditation has also been suggested to stabilize or possibly lengthen the telomeres (Conklin et al., 2018).

Most of the previous studies on the effects of meditation on telomeres have been conducted in patients with cancer (Lengacher et al., 2014; Carlson et al., 2015), infertility (Gautam et al., 2018), depression (Tolahunase et al., 2017) and Alzheimer’s disease (Innes et al., 2018), with only a few investigating the effects of meditation on telomere dynamics in healthy subjects (Hoge et al., 2013; Alda et al., 2016; Le Nguyen et al., 2019; Mendioroz et al., 2020). Recent studies have shown varied results, with some reporting expert meditators to have longer relative TLs (Alda et al., 2016; Dasanayaka et al., 2021), higher telomerase activity (Jacobs et al., 2010; Dasanayaka et al., 2022) and up regulated expression of *hTR* and *hTERT* genes that encode telomerase enzyme (Duraimani et al., 2015) compared to control groups, while some other studies reported that no change occurs in aforementioned telomere characteristics (Sharma et al., 2008; Mendioroz et al., 2020; Schutte et al., 2020). In addition, a recent study showed a positive correlation between the duration of daily meditation and *hTERT* expression, suggesting a potential “dose-dependent” relationship (Contractor et al., 2023). Several studies have indicated a beneficial association between meditation practice and DNA methylation (Lemee et al., 2017; Chaix et al., 2019; Mendioroz et al., 2020; Kripalani et al., 2022). Specially, they showed that as the number of years of meditation practice increases, the levels of methylation decreases and gene expression increases (Lemee et al., 2017). Only a few studies have looked into the effects of meditation on the methylation level of telomere regulatory genes, such as, *hTERT*. Cross-sectional studies have shown that long-term meditators showed epigenetically altered expression of *hTERT* gene compared to controls who have not engaged in meditation practice. Notably, such beneficial effects have mainly been observed in varied populations, and there have been few investigations into the impact of multiple characteristics and outcomes within the same study group. Furthermore, such changes related to telomeres due to effects of meditation were usually reported following short, focused interventions ranging from 4 to 12 weeks (Thimmapuram et al., 2017; Tolahunase et al., 2017; Conklin et al., 2018; Le Nguyen et al., 2019; Puhlmann et al., 2019).

We hypothesized that long-term meditation practice is associated with changes in specific molecular markers of cellular aging and longevity. Hence, the aim of this study was to investigate the relative telomere length, *hTERT* and *hTR* gene expressions, and methylation level in the promoter region of *hTERT* gene in long-term meditators compared to non-meditators. Using this same study cohort, we previously reported that long-term meditators had significantly higher plasma telomerase levels, higher trait mindfulness and improved quality of life (Dasanayaka et al., 2022) and herein, we also build upon our previously published data to investigate the variability of the TL in relation to these multiple molecular and psychosocial variables. This study is a part of a larger ongoing project on physical, social and environmental impacts of meditation.

## 2. Materials and methods

### 2.1. Study design and participants

This observational, case–control study included 30 skilled, long-term meditators from the community who practiced in several meditation centres in Sri Lanka, and 30 non-meditators from the community who were matched for age (±2 years), sex, and educational level. Based on previously published fold change (FC) values of hTERT gene expression, a total sample size of 60 participants: 30 each for meditators and controls, was found to yield an effect size of 0.8 and 80% power for a 0.05 alpha error (Li et al., 2005; Qu et al., 2013). The recruitment process of participants has previously been published (Dasanayaka et al., 2022). Briefly, individuals between the ages of 18–65 years were recruited into this study. The meditators were those who had meditated regularly for more than 6 h a week and had been maintaining a regular meditation practice for 3 or more years. Purposive sampling was used to choose non-meditators (controls) from the same population if they had never or rarely (less than once per 3 months) practiced meditation or other mind–body interventions (Dasanayaka et al., 2022). Participants with any illness or chronic use of medication, smokers and pregnant or breast-feeding women were excluded.

#### 2.1.1. Ethics statement

The study was conducted according to the guidelines of the Declaration of Helsinki and approved by the Ethics Review Committee of Faculty of Medicine, University of Colombo, Sri Lanka (EC-19-067). Recruitment was conducted between August 15, 2020 and December 15, 2021. All subjects provided informed written consent.

### 2.2. Procedures

Potential participants were initially contacted at meditation centers in different parts of the island. Long-term meditators who had achieved pre-identified skill levels as determined by a questionnaire-based scoring system were recruited for the study (Outschoorn et al., 2020). Selected participants of the two groups provided their signed consent upon arriving at the first visit and submitted their blood samples. Uniformity in blood sampling procedures was achieved by conducting the sampling at a consistent time, specifically in the morning between 8.30 and 9.00 am, under similar circumstances for all participants. 5 mL of whole blood was collected into an Ethylenediamine tetraacetic acid (EDTA) tube and immediately centrifuged at 1400 rpm for 10 min at 4°C. Plasma was separated after centrifugation, and the plasma and remaining whole blood were aliquoted into pre labeled 1.5 mL microcentrifuge tubes. Samples were stored at −80°C until further testing, where they were thawed only immediately prior to analysis.

#### 2.2.1. Socio-demographic data

An interviewer-administered questionnaire was used to collect the socio-demographic data including age, sex, educational level, marital status, sleeping hours, work hours, lifestyle habits including alcohol consumption, dietary patterns, and period of physical exercise (hours). The body mass index (BMI) was computed after measuring the subjects’ height and weight with the aid of standard scales. Five-Facet Mindfulness Questionnaire (FFMQ) and World Health Organization Quality of Life (WHO-QOL) questionnaires were used to comprehensively evaluate participants’ mindfulness level and quality of life (Dasanayaka et al., 2022).

### 2.3. Assessments

#### 2.3.1. Relative telomere length measurement

Genomic DNA was extracted from the buffy coat using Wizard® Genomic DNA purification Kit (Promega, United States) according to the manufacturer’s instructions. The extracted DNA was quantified using a Nanodrop 2000 spectrophotometer (Thermo Scientific, Waltham, MA, United States), aliquoted to minimize the freeze–thaw cycles and stored at −20°C until the assays were performed. The relative TL in the peripheral blood leukocytes was determined via quantitative PCR (qPCR) based technique that compares telomere repeat sequence copy number (T) with a reference single copy-gene (*β*-globin gene) copy number (S) as previously described (Laboratory, n.d.). The DNA concentration of all samples was adjusted to 5 ng/uL prior to performing the assay. To measure the relative TL, quantitative real-time polymerase chain reaction (RT-PCR) was performed using Absolute Human Telomere Length Quantification qPCR Assay Kit (ScienCell, United States). In brief, two qPCR reaction mixers were prepared per sample; one for the single copy reference primer set and the other one for the telomere primer set. Assays proceeded for 1 cycle at 92°C for 10 min, followed by 32 cycles at 95°C for 20 s, 52°C for 20 s and 72°C for 45 s. Reference DNA sample from Absolute Human Telomere Length Quantification qPCR Assay kit, and the aforementioned process was followed to generate the reference sample TL for each of the PCR plates. For each sample, the comparative cycle value of the number of telomere repeats and *β*-globin gene copies was obtained.

Relative TL of the target sample to the reference sample was calculated according to the comparative 2^−ΔΔCq^ (Quantification Cycle Value) method. The total TL of the target sample was obtained in kilobases via multiplying the reference sample TL by the quantification cycle value.

qPCR runs were conducted on a Rotor Gene Q Real-Time PCR System (Applied Biosystems). Each run consisted of an equal number of cases and controls and included negative control wells. Further, all the assays were performed by one person to avoid handling errors and were conducted in the same laboratory to prevent impacts of the local factors such as humidity. Prior pilot studies and validation experiments supported the reliability of duplicate reactions in achieving robust and reproducible results within the scope of this study. Comprehensive quality control measures and standardized protocols further ensure the validity and reliability of the obtained data.

#### 2.3.2. Quantification of gene expression

Whole blood was subjected to RNA extraction by using SV Total RNA Isolation System (Promega, United States) according to manufacturer’s recommendations. For each sample, 1 μg of RNA was subjected to reverse transcription using GoScript^™^ Reverse Transcription System (Promega, USA) to synthesize cDNA. Reverse transcriptase qPCR was performed on 50 ng of the obtained cDNAs by using Quantitech^®^ SYBR^®^ Green PCR master mix, employing the primers for *hTR* and *hTERT* genes detailed elsewhere (Duraimani et al., 2015). Primer sequences are detailed in Table 1. The RT-PCR conditions were optimized as reported in Dasanayaka et al. (2022). Briefly, RT-PCR reactions contained 10 μL of 2 × Quantitech SYBR Green RT-PCR master mix, 2 μL of primers, 50 ng of sample cDNA and Nuclease free water to complete the reaction volume to 20 μL. The thermal cycling profile consisted of 10 min initial denaturation at 95°C (1 cycle), 20 s denaturation at 95°C, 20 s annealing at 60°C and 45 s extension at 72°C followed by 45 cycles and 1 min hold at 60°C. Relative quantification measures the level of target gene cDNA (*hTERT* and *hTR*) relative to the level of *GAPDH* reference gene cDNA. The relative gene expression level was measured using the 2^−ΔΔCt^ method (Rao et al., 2013).

**Table 1:** Primer sequences.

Gene	Forward primer	Reverse primer
*hTR*	5′-GGTGGTGGCCATTTTTTGTC-3	5′-CTAGAATGAACGGTGGAAGGC-3′
*hTERT*	5′-ACGGCGACATGGAGAACA A-3′	5′-GGGTCCTGAGGAAGGTTTTC-3′
*GAPDH*	5′-CAATGACCCCTTCATTGACC-3′	5’-GAAGATGGTGATGGGATTTC-3′

#### 2.3.3. DNA methylation assay

Genomic DNA was treated with bisulphite using the MethylEdge™ Bisulfite conversion system (Promega, United States) in accordance with the manufacturer’s instructions. Thirty nine CpG sites of the *hTERT* promoter, extending 500 bases upstream of the transcriptional start site to 500 bases downstream of the translational start site, were examined for methylation. The primers targeting promoter region of *hTERT* gene for amplifying bisulphite-modified DNA were: forward primer 5’-CTACCCCTTCACCTTCCAA-3′, and reverse primer 5′-GTTAGTTTTGGGGTTTTAGG-3′. Thermocycling conditions were an initial step at 95°C (5 min), then 45 cycles of the following steps: 95°C (60 s), 59°C (30 s) and 72°C (35 s). 3 μL of the PCR product was visualized on a 1.5% agarose gel. Each PCR product was sequenced using the *hTERT* forward primer on SeqStudio™ Genetic Analyzer System with SmartStart, (Thermofisher, United States). Sequence reads were visualized by Bio Edit Sequence Alignment Editor software (Alzohairy, 2011). Sequences with noisy data background were trimmed and introduced to the BiQ analyzer software which displays the methylated sites and unmethylated sites separately (Akgün et al., 2022).

### 2.4. Statistical analysis

The Shapiro–Wilk test was used for testing the normality of the data (Ghasemi and Zahediasl, 2012). Discrete variables are presented as frequencies or percentages and the continuous variables as mean ± SD. Comparison of continuous variables such as relative TL, gene expressions, and socio-demographic factors including age, BMI, and sleeping hours were done by independent t-test whereas comparison of categorical variables such as sex, education level, married percentage, number of non-vegetarians, alcohol consumers, and hours of exercise were carried out with chi-square test. Using Cohen’s d test, the variations in effect sizes on TL between meditators and non-meditators were evaluated. Pearson correlation was used to determine the bivariate relationships between TL and socio-demographics, and previously published psychological variables, i.e., mindfulness (FFMQ questionnaire) (Baer et al., 2008) and quality of life (WHO-QOL questionnaire) (Kumarapeli et al., 2006). A multivariate linear regression model with a backward elimination approach was used to analyze relationships between telomere length and socio-demographic factors and psychological outcomes. This model took into account the socio-demographic and psychological variables that produced significant results in the correlation analysis.

Independent t-test was used to compare the fold change in gene expression between meditators and controls, demonstrating a notable dissimilarity in the gene expression levels and suggesting that meditation may have a regulatory effect on gene expression.

Sanger sequencing analysis was used to assess the level of methylation at specific CpG sites in the promoter region of *hTERT* gene. The average percentage of the methylation over all the promoter CpG sites was calculated. Considering the median methylation level of the control group, methylation percentage was categorized into two groups. If the overall methylation was greater than the median methylation of the control group at the promoter region, this was considered as a high methylation category whereas the lower overall methylations relative to the median methylation of the control group was considered as the low methylation category. Accordingly, methylation levels of *hTERT* gene among meditators and non-meditators were compared after adjusting for age, sex and diet and presented as Odds ratios with 95% confidence intervals.

The statistical analyzes were conducted using IBM SPSS (Version 23.0), and significance was determined by value of *p*-values than 0.05.

## 3. Results

### 3.1. Characteristics of study population

Table 2 summarizes the baseline characteristics of the study population which have been described before (Dasanayaka et al., 2022). Briefly, nineteen individuals in each group of 30 participants (63.34%) were male with an average age of 43 years. All the participants were of Sinhalese ethnicity and only three participants were Christians while the religion of the remainder was Theravada Buddhism. Seven meditators and eight controls had consumed alcohol occasionally (Drinking alcohol only at attendance at a party is defined as occasional alcohol consumption) and 8 meditators and 13 controls had exercised for more than 1 hour per week. The mean duration that meditators had practiced meditation over their lifetime was 6.8 years (±3.27) and the mean period of daily meditation reported by the meditators was 5.82 h (±3.45). They reported regularly practicing one or more of a particular type of meditation technique which included loving-kindness, breathing and body scanning meditation. The baseline characteristics did not differ significantly between the meditator and non-meditator groups.

**Table 2:** Socio-demographic and health characteristics of the study population.

	Sex (male)^a^	Age (years) (mean ± SD)^a^	Married %	Educational level – Tertiary education^*^	Educational level – secondary education^**^	Body mass index (BMI)	Alcohol^b^	Smoking	Non-vegetarian diet	Sleeping hours per day	Exercise (>1 h/week)
Meditators	19/30 (63.34%)	43.83 ± 9.92	19/30 (63.34%)	24/30 (80%)	6/30 (20%)	26.5 ± 5.23	7/30 (23.3%)	0/30 (0%)	29/30 (96.67%)	6.27 ± 1.56	8/30 (26.67%)
Non-meditators	19/30 (63.34%)	43.51 ± 9.92	14/30 (46.6%)	24/30 (80%)	6/30 (20%)	23.39 ± 2.61	8/30 (26.67%)	0/30 (0%)	30/30 (100%)	6.22 ± 1.92	13/30 (43.34%)
Significance	*p* = 1	*p* = 0.897	*p* = 0.407	*p* = 1	*p* = 1	*p* = 0.227	*p* = 1	*p* = 1	*p* = 1	*p* = 0.987	*p* = 0.1

a Matched variables.

b Consume alcohol occasionally (Drinking alcohol only at attendance at a party is defined as occasional alcohol consumption).

^*^Tertiary education-Certificate/Diploma/Degree/post graduate degree, ^**^Secondary education-from grade 6 to 13 (GCE Advanced Level).

### 3.2. Telomere length

**Figure 1:**
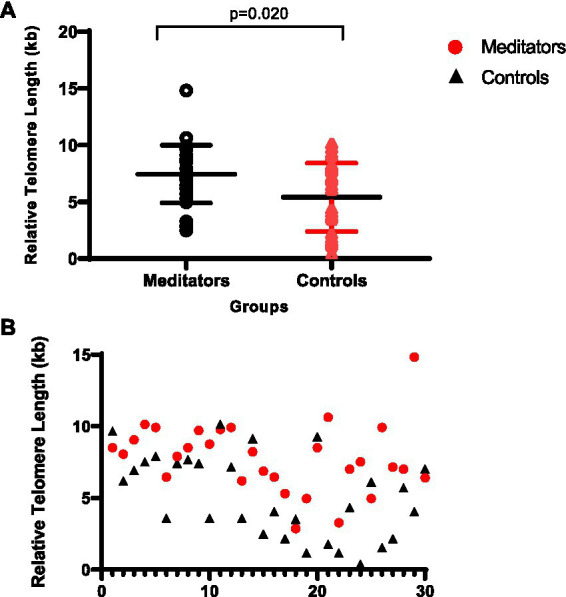
**(A)** Comparison of relative telomere length between meditators and non-meditators. Telomere length was significantly higher in meditators compared to the age-, sex-, and education-level matched controls, *p* = 0.020. Horizontal lines represent the mean ± standard deviation of the individuals’ telomere length. **(B)** Comparison of relative telomere length distributions: Meditators vs. Controls.

Variability of the TL between the two groups is shown in Figure 1. The long-term meditators had a significantly longer relative TL (mean ± SD = 7.35 ± 2.53 kb) compared with the control group (mean ± SD = 5.57 ± 3.15 kb) (*p* = 0.020, Cohen’s d = 0.61).

### 3.3. Relationship of telomere length with socio-demographics, psychological variables and meditation practice

**Figure 2:**
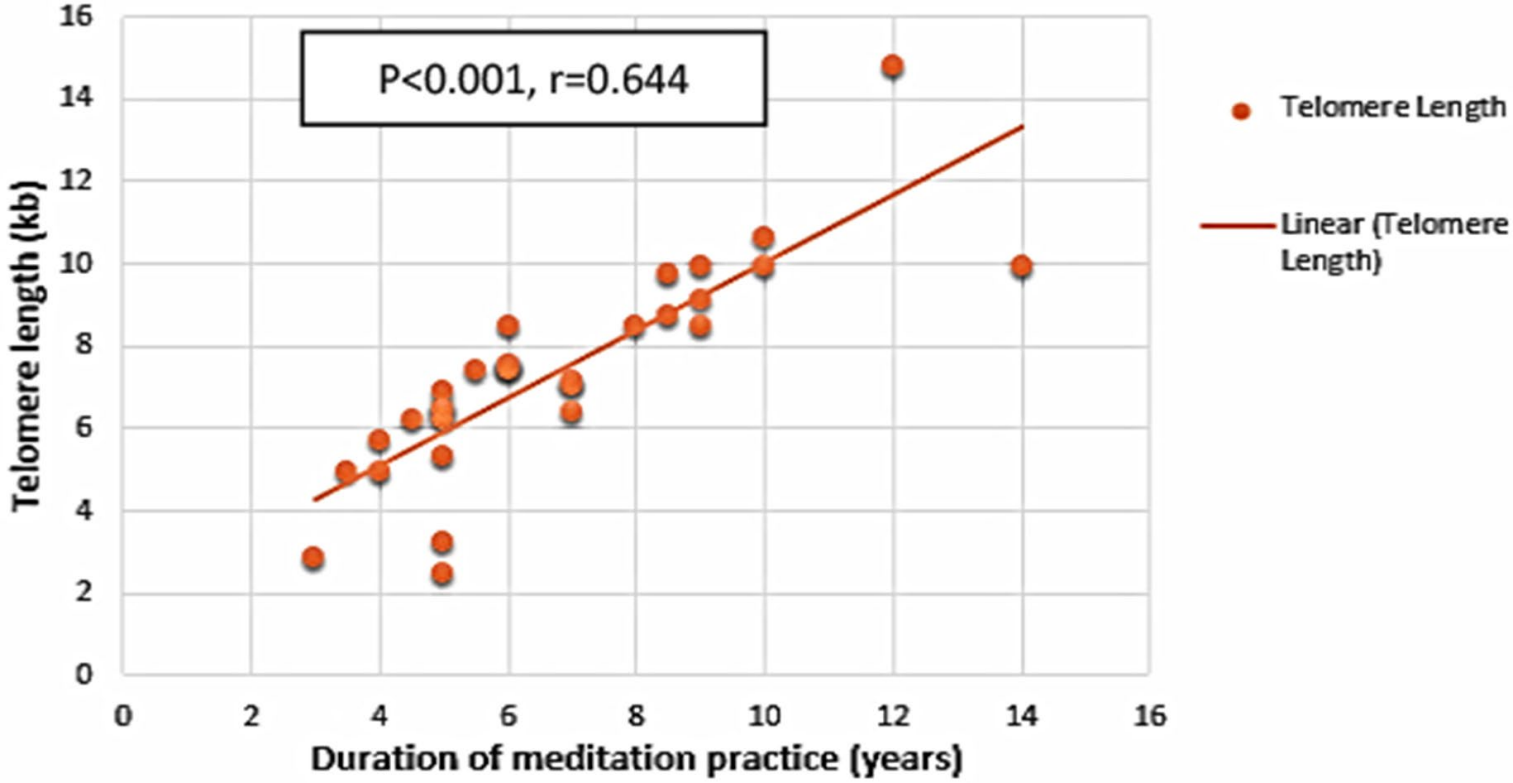
Duration of meditation and telomere dynamics. Scatter plot with linear correlation showing the relationship between duration of meditation practice and telomere length in all long-term meditators (*r* = 0.644, *p* < 0.001).

Age was the only sociodemographic factor that significantly correlated with TL (*r* = −0.675, *p* < 0.001). In addition, there was a strong positive correlation between the meditators’ total time spent practicing meditation and the length of their telomeres (*r* = 0.644, *p* < 0.001) (Figure 2). Notably, the TL was not associated with any of the other variables including BMI, number of working hours per day, number of hours spent outside, number of sleeping hours per day and the number of hours of exercise per day. Further, positive correlations were observed between TLs and psychological variables such as acting with awareness (*r* = 0.410, *p* = 0.001) and non-reactivity (*r* = 0.309, *p* = 0.017) in mindfulness subscales and physical health (*r* = 0.438, *p* = 0.001) and social relationship (*r* = 0.282, *p* = 0.030) in quality-of-life subscales.

We performed a regression analysis to identify factors which could predict telomere length. Age, the duration of meditation practice, and all psychological factors that had a significant correlation with TL were included in a backward stepwise regression model. Accordingly, the TL was significantly influenced by age (beta = −0.614, *p* = 0.001) and the duration of meditation practice (beta = 0.389, *p* = 0.003). The quality-of-life subscale for social relationships and the mindfulness subscale for non-reactivity did not significantly contribute to the final model.

### 3.4. *hTR* and *hTERT* gene expression

**Figure 3:**
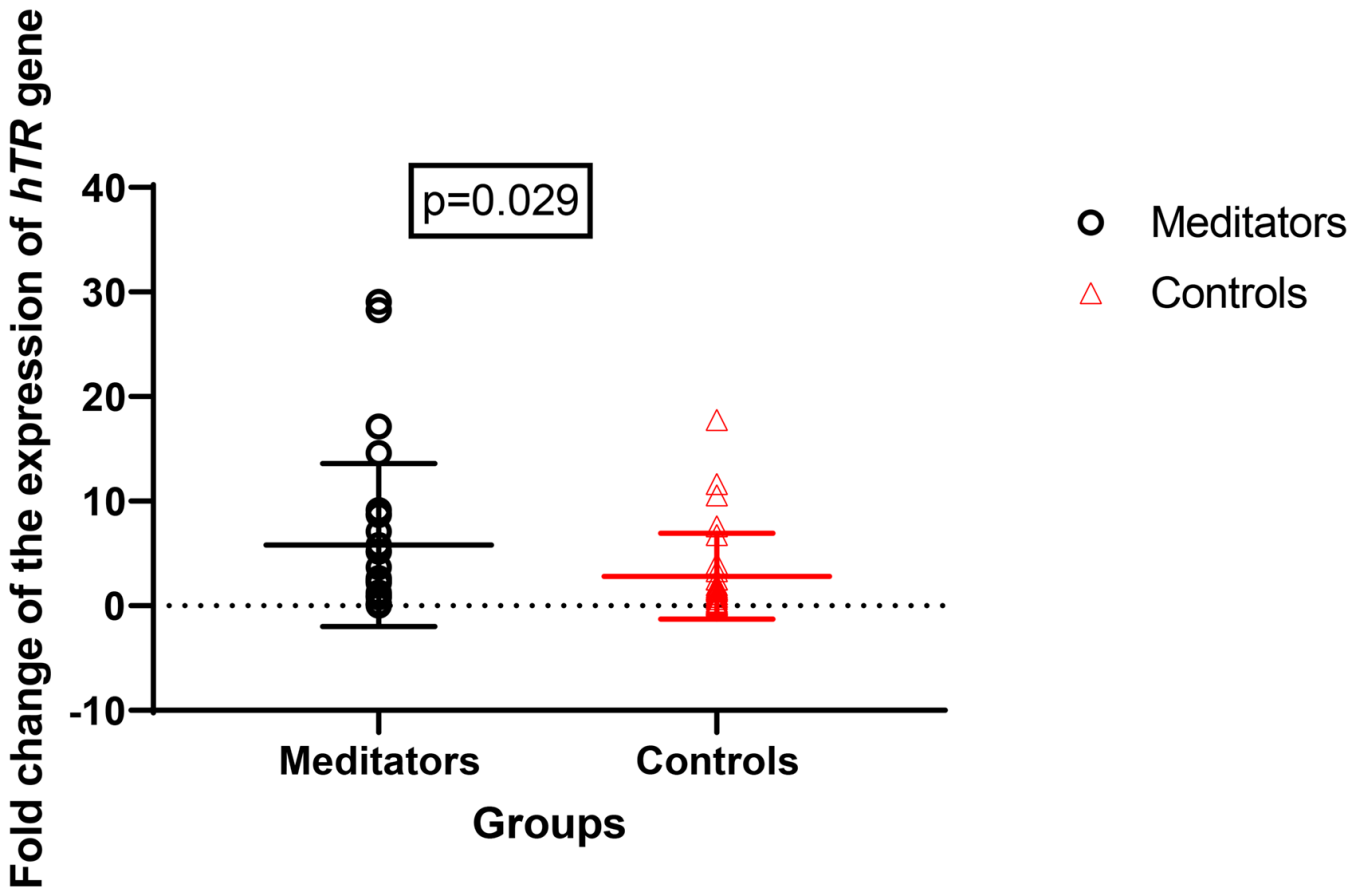
Comparison of *hTR* gene expression between meditators and non-meditators. Expression of *hTR* (*p* = 0.029) gene was significantly higher in meditators compared to age-, sex-, and educational-level matched controls. Horizontal lines represent the mean ± standard deviation of the individuals’ gene expression.

**Figure 4:**
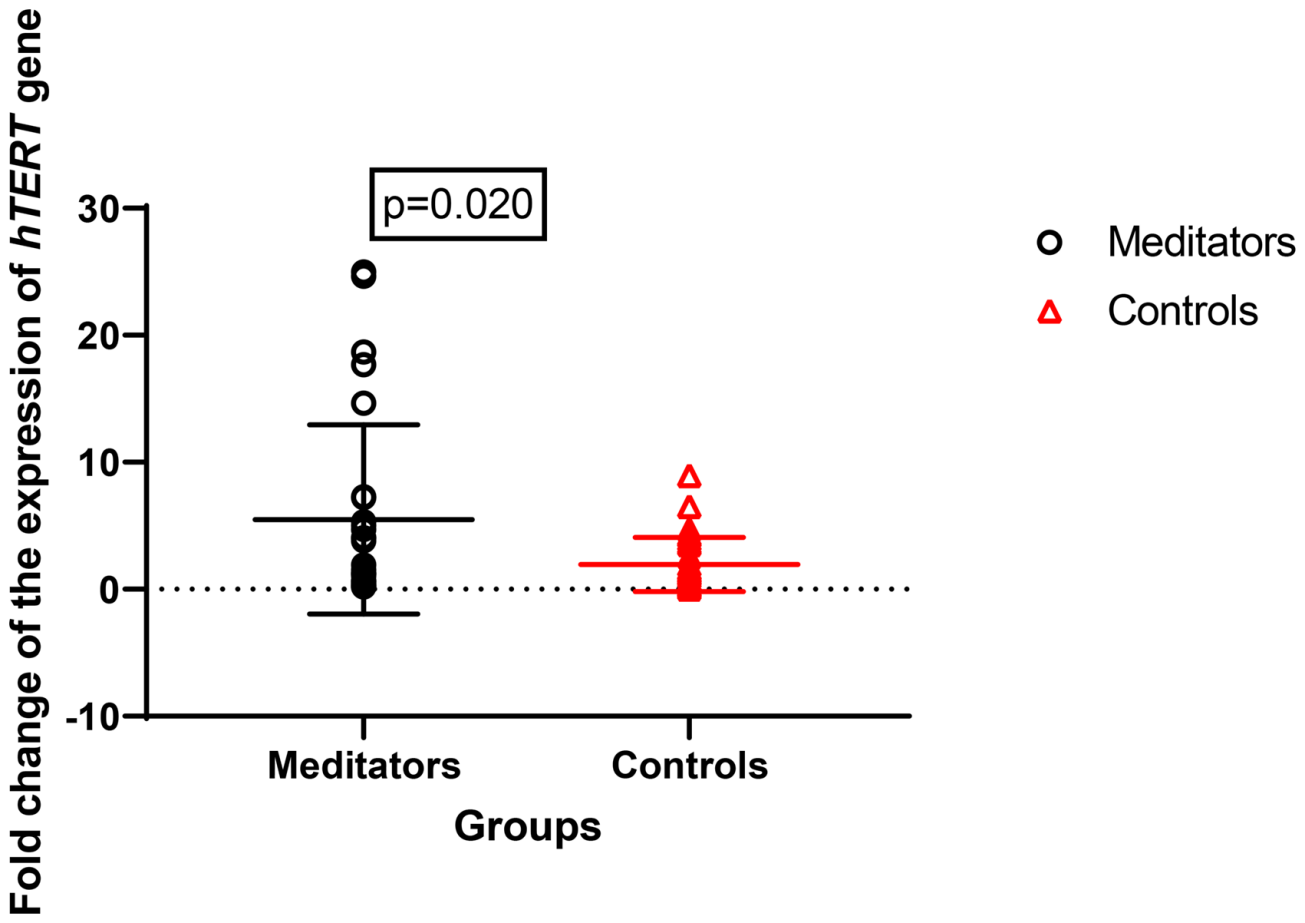
Comparison of *hTERT* gene expression between meditators and non-meditators. Expression of *hTERT* (*p* = 0.020) gene was significantly higher in meditators compared to age-, sex-, and educational-level matched controls. Horizontal lines represent the mean ± standard deviation of the individuals’ gene expression.

Expression of the *hTERT* gene (meditators: mean ± SD = 8.67 ± 13.28, control: mean ± SD = 2.82 ± 4.11, FC = 1.3, *p* = 0.020) and *hTR* gene (meditator: mean ± SD = 10.53 ± 22.75, control: mean ± SD = 1.96 ± 2.13, FC = 1.4, *p* = 0.029) showed significant up-regulation in long-term meditators compared to controls (Figures 3, 4).

### 3.5. Methylation of *hTERT* gene

The methylation status of the *hTERT* promoter region was assessed from −500 nucleotides relative to the transcriptional start site to +500 nucleotides. The promoter region of the *hTERT* gene had overall higher methylation levels in the meditators than in non-meditators after controlling for age, sex and diet (meditator: mean ± SD = 62.64 ± 9.7%, control: mean ± SD = 76.75 ± 9.73%, Odds ratio-12.640, 95% CI, 0.366 to 4.614; *p* < 0.001). Further analysis of methylation levels at each of the 39 CpG sites in the promoter region of the *hTERT* gene on chromosome 5 from position 1,294,800 to 1,295,065 is presented in Table 3. Significantly lower methylation levels were observed in the long-term meditators compared to controls at 12 of the CpG sites even after adjusting for age, sex, and diet (Table 3).

**Table 3:** Comparison of *hTERT* gene methylation at each CpG site between the long-term meditators and the controls.

	Cases unmethylated C	Controls unmethylated C	Location	*p* value
CpG 1	30	5	5:1295061	<0.001*
CpG 2	30	8	5:1295059	<0.001*
CpG 3	19	17	5:1295053	0.029*
CpG 4	0	0	5:1295047	0.998
CpG 5	0	18	5:1295044	0.997
CpG 6	16	12	5:1295040	0.374
CpG 7	0	0	5:1295038	1.000
CpG 8	30	30	5:1295029	1.000
CpG 9	15	10	5:1295027	0.221
CpG 10	0	0	5:1295023	1.000
CpG 11	14	10	5:1295021	0.183
CpG 12	25	2	5:1295006	<0.001*
CpG 13	25	3	5:1295000	<0.001*
CpG 14	0	0	5:1294985	1.000
CpG 15	25	5	5:1294983	<0.001*
CpG 16	0	0	5:1294978	1.000
CpG 17	30	21	5:1294974	0.998
CpG 18	0	0	5:1294972	1.000
CpG 19	0	0	5:1294969	1.000
CpG 20	30	20	5:1294967	0.998
CpG 21	0	0	5:1294957	1.0000
CpG 22	14	9	5:1294951	0.027*
CpG 23	6	2	5:1294945	<0.001*
CpG 24	5	2	5:1294941	<0.001*
CpG 25	12	0	5:1294936	0.998
CpG 26	0	0	5:1294918	1.000
CpG 27	1	0	5:1294913	0.999
CpG 28	1	0	5:1294909	0.998
CpG 29	0	0	5:1294905	1.000
CpG 30	0	0	5:1294897	1.000
CpG 31	18	3	5:1294880	<0.001*
CpG 32	0	0	5:1294870	1.000
CpG 33	19	11	5:1294867	0.047*
CpG 34	0	0	5:1294863	1.000
CpG 35	0	0	5:1294854	1.000
CpG 36	0	0	5:1294844	1.000
CpG 37	14	11	5:1294835	0.004*
CpG 38	0	0	5:1294819	1.000
CpG 39	0	0	5:1294802	1.000

*p-value for the difference between meditators and controls was analyzed by logistic regression and was adjusted by age, sex and diet *α* = 0.05.

As expected, methylation level of the promoter region of *hTERT* gene and the expression of the *hTERT* gene showed a significant negative correlation (*r* = −0.589, *p* = 0.001). Similarly, there was a significant negative relationship between methylation level of the promoter region of *hTERT* gene and duration of meditation practice (*r* = −0.580, *p* = 0.001).

## 4. Discussion

This study examined the associations of several telomere related indices with the practice of long-term meditation. Our findings demonstrate that long-term meditators have significantly longer relative TLs, and that the duration of meditation practice and age are predictors of relative TL. Further, our results showed that individuals who practice long-term meditation have significantly higher expression of *hTERT* and *hTR* genes compared to non-meditators, and also lower levels of methylation in the promoter region of the *hTERT* gene. We also demonstrated that increasing years of practice of meditation is associated with lower levels of methylation in the promoter region of *hTERT* gene.

In a background of increasing knowledge on telomere dynamics (Gruber et al., 2021), these results add to the understanding of underlying mechanisms of how the regular practice of meditation may positively affect the pace of aging, as well as the onset and progression of many age-related diseases such as cardiovascular disease, malignancies, dementia and osteoporosis. The telomerase enzyme helps to maintain the TL. The activity of telomerase enzyme is known to be primarily dependent on transcriptional regulation of *hTERT* gene (Riethman, 2008) while variations in *hTR* gene resulting in altered telomerase RNA template has also been shown to reduce telomerase activity (Theimer and Feigon, 2006). Several previous studies on psychosocial genomics indicate that distinct gene expression changes could be induced not only by physical influences but also through social, psychological and cultural components (Pinaud, 2004; Ray, 2004; Mccutcheon, 2010). Methylation is one of the main factors influencing the expression of *hTERT* gene. Our findings show that long term practitioners of meditation have lower levels of methylation in the promoter region of the *hTERT* gene and hence it is effectively expressed. As a result of the transcriptionally active *hTERT* gene, telomerase enzyme production rises which in turn reduces the shortening of telomere length.

We have previously reported that meditation improves mindfulness and quality of life (Duraimani et al., 2015). Increased stress causes cells to produce more oxidative agents like reactive oxygen species (ROS) and nitrogen oxides (Krishna et al., 2015) which could reduce telomere length by damaging telomeres. Another sub-study conducted by our research group and reporting on these same participants, found that the long-term meditators had lower levels of nitric oxide and higher antioxidant capacity compared to the controls (Thambyrajah et al., 2022).

Accordingly, the findings suggest that meditation reduces stress both at a psychological and molecular level, thereby contributing to reduce the shortening of telomere length. Our current and previous (Duraimani et al., 2015) data has shown that longer duration of meditation practice is associated with both longer telomere lengths and higher levels of telomerase enzyme. While the exact drivers still remain unclear, meditation is likely to contribute to cellular longevity through these multiple mechanisms which increase telomere length and thus, maintain telomere integrity.

Rather than recruiting experienced meditators considering only one criterion, i.e., duration of meditation practice, we identified those who had gained the necessary skill levels using a specially designed, questionnaire-based screening tool (Outschoorn et al., 2020) which would have contributed to the significant findings of this study, even with a limited sample size. Interestingly, our results showed positive effects of meditation in long-term meditators who had been practicing different types of meditation techniques, which may suggest that the beneficial effects of meditation may not primarily be dependent on the specific technique, and should ideally be confirmed with a larger multi-arm study comprising practitioners of different techniques. Moreover, similar results for TL, expression of *hTERT* and *hTR* genes, and methylation level of the promoter regions of *hTERT* gene have been obtained for different meditation practices including mindfulness meditation, loving kindness meditation, and Zen meditation by previous research studies (Singh et al., 2009; Jacobs et al., 2010; Hoge et al., 2013; Alda et al., 2016; Le Nguyen et al., 2019; Mendioroz et al., 2020).

We further observed that the relative TL was negatively associated with age, which suggests that while physiological shortening of telomeres associated with aging cannot be prevented, practicing meditation may decrease the rate of reduction of TL. Most of the meditators had completed their tertiary education which indicates that individuals with higher education levels are more likely to practice meditation and/or gain skill levels. Socio-demographic features other than age, did not show any relationship with the relative TL in our study. A previous study on the association of loving-kindness meditation with telomeres has shown that the association between meditation and the relative TL is stronger in women than men (Hoge et al., 2013). However, we did not analyze this association due to unequal sex sampling with a higher percentage (63.34%) of males in the current study population. We recruited skilled meditators and controls who were determined to be healthy *via* a mini-interview. Although the therapeutic advantages of meditation are more alluring, a segment of the academic community has started to look at the impact of meditation on non-clinical groups with noticeable and encouraging findings which led to the selection of a broader population.

The current study has several limitations. The major challenge was addressing multiple confounders which could affect the outcome variables. Various environmental and lifestyle factors may influence the *hTR* and *hTERT* gene methylation and expression and affect TLs in similar ways to meditation. Furthermore, this study is cross-sectional in nature, which should be considered as a limitation, as it restricts the ability to establish causal relationships of the findings. Confirmation of the effect of long-term meditation on TL using a larger sample in a longitudinal study is recommended. A sensitivity analysis based on the type of meditation was not conducted due to many meditators practicing two or more meditation techniques, which limited our ability to isolate the effects of individual practices. The limited sample size prevented us from performing a sub-analysis based on age groups, which would have provided a more precise picture of the telomere dynamics. This study could not determine how long these effects lasted without practicing meditation and accordingly could not define the transient and stable changes over time. This may provide an intriguing line of future research.

## 5. Conclusion

The findings of this case–control study suggest that long-term meditation practice has beneficial effects on the TL, expression of *hTERT* and *hTR* genes and the methylation level of the promoter regions of *hTERT* gene with the potential to delay cellular aging. This study contributes to the growing body of research exploring the effects of meditation practices on molecular changes. While we specifically focused on evaluating one of the hallmarks of cellular aging, our findings provide intriguing insights into the potential impact of long-term meditation practice. This approach can be utilized to systematically interrogate signals associated with molecular changes induced by meditation practices. As a result, our study strengthens the genetic and epigenetic evidence supporting the incorporation of meditation practice into regular lifestyle of person and complementary therapeutic approaches. Further investigations that encompass multiple cellular aging hallmarks are warranted to gain a more comprehensive understanding of the potential benefits of meditation. Confirmation of the effect of long-term meditation on TL using a larger sample in a longitudinal study is recommended.

## Data availability statement

The data presented in the study are deposited in Figshare repository. DOI: 10.6084/m9.figshare.23537952 Link: https://figshare.com/s/eb446a9f62afe25cae3e.

## Ethics statement

The studies involving human participants were reviewed and approved by Institutional Ethics Committee of Faculty of Medicine, University of Colombo. The patients/participants provided their written informed consent to participate in this study.

## Author contributions

NSa and NSi: study conception and design. ND: data collection, analysis, and interpretation of results. ND, NSi, and NSa: draft manuscript preparation. All authors contributed to the article and approved the submitted version.

## Funding

This work was supported by a grant from the Accelerating Higher Education Expansion and Development (AHEAD) Operation of the Ministry of Higher Education funded by the World Bank (Grant No. 6026-LK/8743-LK).

## Conflict of interest

The authors declare that the research was conducted in the absence of any commercial or financial relationships that could be construed as a potential conflict of interest.

## Publisher’s note

All claims expressed in this article are solely those of the authors and do not necessarily represent those of their affiliated organizations, or those of the publisher, the editors and the reviewers. Any product that may be evaluated in this article, or claim that may be made by its manufacturer, is not guaranteed or endorsed by the publisher.
